# How to reveal disguised paternalism: version 2.0

**DOI:** 10.1186/s12910-021-00739-8

**Published:** 2021-12-28

**Authors:** Niels Lynøe, Ingemar Engström, Niklas Juth

**Affiliations:** 1 Centre for Healthcare Ethics, Karolinska Institute, 171 77 Stockholm, Sweden; 2 University Health Care Research Centre, Faculty of Medicine and Health, Örebro University, Örebro, Sweden

**Keywords:** Physician assisted suicide, Disguised paternalism, Hard and soft paternalism, Value impregnated factual claims

## Abstract

**Background:**

We aim to further develop an index for detecting disguised paternalism, which might influence physicians’ evaluations of whether or not a patient is decision-competent at the end of life. Disguised paternalism can be actualized when physicians transform hard paternalism into soft paternalism by questioning the patient’s decision-making competence.

**Methods:**

A previously presented index, based on a cross-sectional study, was further developed to make it possible to distinguish between high and low degrees of disguised paternalism using the average index of the whole sample. We recalculated the results from a 2007 study for comparison to a new study conducted in 2020. Both studies are about physicians’ attitudes towards, and arguments for or against, physician-assisted suicide.

**Results:**

The 2020 study showed that geriatricians, palliativists, and middle-aged physicians (46–60 years old) had indices indicating disguised paternalism, in contrast with the results from the 2007 study, which showed that all specialties (apart from GPs and surgeons) had indices indicating high degrees of disguised paternalism.

**Conclusions:**

The proposed index for identifying disguised paternalism reflects the attitude of a group towards physician assisted suicide. The indices make it possible to compare the various medical specialties and age groups from the 2007 study with the 2020 study. Because disguised paternalism might have clinical consequences for the rights of competent patients to participate in decision-making, it is important to reveal disguised hard paternalism, which could masquerade as soft paternalism and thereby manifest in practice. Methods for improving measures of disguised paternalism are worthy of further development.

## Background

Medical paternalism means that, for example, a physician finds that a patient does not understand her or his own best interests—e.g. the beneficial consequences of an offered treatment that the patient declines—and then makes the decisions on behalf of the patient [1, 2]. If such a patient is actually competent, the action is referred to as hard paternalism. In practice the physician might inform the patient in a manner such that the patient cannot decline the offered treatment, and the physician can motivate such an action by finding the patient to be incompetent and hence transforming hard paternalism to apparently soft paternalism [3].

If a physician finds that a patient’s preference is in conflict with the physician’s basic values about what is harmful (e.g., it contradicts the physician’s interpretation of the dictum “do no harm”, also called the non-maleficence principle), then the physician might try to persuade the patient to change their mind about the things the physician thinks are harmful. In the attempt to persuade the patient, the physician might use different strategies, from soft nudges to decisions based on hard paternalism [3–5].

The patient-physician relationship, however, is supposed to be based on patient-centred care with respect for the patients’ rights to participate in decision-making, which, in turn, is based on the principle of autonomy [1]. In principle, patient autonomy means that there is no reason for a physician to make decisions about what is in a competent patient’s best interest [4]. There might, however, be circumstances where hard paternalism is justified, e.g. in cases where the patient is declining life-saving treatment due to unbearable pain [6].

If so, it might be justifiable to make preliminary decisions on behalf of such a patient, at least until the patient’s pain is relieved [1, 2]. Moreover, if a patient is considered not to be fully competent and a physician in such a situation makes surrogate decisions in the best interest of the patient, this is called *soft paternalism*. As indicated, this is not to say that hard paternalism may never be justified, but rather that it is much more difficult to justify than soft paternalism simply because in hard paternalism the patient is judged to be competent enough to make up their own mind [1, 2].

Even though there are differences among countries and cultures, a general tendency is that hard paternalism is considered outdated and inappropriate in health care [7]. The physician often still knows and understands more than the patient about medical matters, but this does not imply that they always know what is in the patient’s best interest. The patient might have preferences and values that differ from those of the physician. For example, if a patient suffering from prostate cancer prefers a half year shorter lifetime with higher quality of life to a longer life with lower quality of life, there might be a genuine value conflict with the physician, who might prefer to have it the other way around [8]. In the end, however, after an adequate amount of information—e.g. about the consequences of different treatment options—has been acquired in order to prevent misunderstandings, a physician is supposed to respect a competent patient’s values and preferences [6, 8].

Because physicians today are generally aware that hard paternalism implies disrespecting and wronging a competent patient, physicians’ efforts to influence the decision-making of patients is often unintentional. Instead, physicians may covertly express tacit values that unconsciously influence the provided information to the patient or the evaluation of whether or not the patient is competent [3]. A Swiss study about physicians’ personal values indicated an increased risk among some physicians for arbitrariness when determining a patient’s decision-making capacity [9, 10]. There are also indications that whether a palliative physician offers a patient palliative sedation depends on that physician’s own personal values, including religious values [3, 4, 9–11]. Letting one’s own values inadvertently influence evaluations of patients, especially with regard to decision-making competency, can result in misclassifying a patient as incompetent. In this context we refer to this process as *disguised paternalism*.

Disguised paternalism might, however, also represent other disguised aspects such as disguised values and disguised self-interest. Disguised self-interest might be actualized particularly in countries where there is no legal room for conscientious objections, such as in Sweden. These three entities—disguised paternalism, disguised values, and disguised self-interest—are probably interwoven and difficult to separate, and we have accordingly focused on disguised paternalism.

An example of disguised paternalism was examined in a study from 2007 about Swedish physicians’ attitudes towards physician assisted suicide (PAS) in which the physicians were asked to prioritize among different arguments for or against PAS [12]. In the context of this study, it was possible to develop an index of disguised paternalism [13]. In the present paper, we report the results of our 2020 repeat of the 2007 study and our attempt to further develop and examine this index of disguised paternalism as well as to analyse its consequences.

## Methods

Both the 2007 study and the 2020 study were performed as cross-sectional surveys and were conducted as postal questionnaires with a similar structure [12, 14]. In the 2007 study the main question was the participants’ attitude towards PAS, whereas in the 2020 study we included two additional questions regarding whether or not the participant would like to be offered PAS themselves and whether or not they were prepared to actually prescribe the needed drugs [14]. Moreover, in the 2007 survey the participants were presented with 10 fixed response arguments [12] that were either in favour of PAS or against PAS—see Box 1. The participants were asked to prioritize the arguments according to their own professional opinion about which arguments were most persuasive to them. In the 2020 survey, the number of fixed response options was reduced to four arguments—see Box 1 (marked with bold text). Two of these arguments favoured PAS—(a) based on the autonomy principle by itself and b) based on the reasoning that the autonomy principle should overrule the non-maleficence principle—and two arguments against PAS—(a) the patients do not know their own best interests and b) that the non-maleficence principle should overrule the autonomy principle. As can be seen, one of the arguments was openly paternalistic (the patients do not know their own best interests) and one argument was supposed to represent *disguised paternalism* (the non-maleficence principle should overrule the autonomy principle), while the remaining two arguments supported the autonomy principle [12, 14]. The arguments that were included in the 2007 study and excluded in the 2020 study were, for example, ‘Palliative care is lacking in your region’ as a pro PAS argument and ‘Palliative care in your region is well established’ as an argument against PAS [12]. Other arguments that were excluded were ‘Alternative actions patients might use are painful’ and ‘Risk of pressure on patients who do not want to become a burden to relatives’—see Box 1. Because these arguments were less relevant when assessing pros and cons of the autonomy principle versus paternalism and due to risk of a lower response rate if the questionnaire became too long, we focused on arguments relevant to the autonomy principle and whether respect for autonomy should precede the non-maleficent principle.

**Box 1:** The arguments that were provided in the 2007 study (n = 10)—including 5 pro and 5 cons—and in the 2020 study (n = 4)—including 2 pro and 2 cons—marked with bold letters

*Pro-arguments*:
**Respect for patients’ autonomy**
The purpose is to minimize suffering, not shorten life
**The patient’s autonomy takes precedence over the non-maleficence principle**
Alternative actions that patients might use are painful
Palliative care is lacking in your region
*Contra-arguments*:
**Patients do not know their own good in such cases**
Risk of jeopardizing trust in physicians
**The non-maleficence principle takes precedence over patients’ autonomy**
Risk of pressure on patients who do not want to become a burden to relatives
Palliative care in your region is well established

In order to compare the 2020 study with the 2007 study, we re-calculated the 2007 study regarding how the participants evaluated the importance of the four arguments in terms of being very important, rather important, rather unimportant, or not important at all. The results are presented as proportions of those who stated that an argument was very important or rather important. We also asked the participants to identify which of the arguments they found to be the most important. The proportion of the prioritized arguments is presented with 95% confidence intervals (CIs), and CIs that were not overlapping were considered significant (p < 0.05) as if a hypothesis test had been conducted—see Table 1.

**Table 1:** The average proportions of the six specialties who stated that arguments were (very or rather) important

Arguments	The 2007 study		The 2020 study	
	Important: Yes % (95% CI)	Prioritized (n = 338)	Important: Yes %	Prioritized (n = 572)
Respect for patient’s autonomy	(n = 661) 87% (84–90)	55% (50–60)	(n = 714) 88% (86–90)	50% (46–54)
Patients do not know their own good	(n = 679) 74% (71–77)*	7% (4–10)	(n = 702) 59% (55–63)*	4% (2–6)
Autonomy principle precedes non-maleficence principle	(n = 656) 54% (50–58)	3% (1–5)	(n = 699) 57% (53–61)	6% (4–8)
Non-maleficence principle precedes autonomy principle	(n = 677) 63% (59–67)	35% (30–40)	(n = 697) 68% (65–71)	40% (36–44)

The participants were also asked to prioritize between the four arguments—also in both the 2007 study and the 2020 study, respectively. In order to compare the 2007 study with the 2020 study, palliative care physicians were not included in the 2020 study. An * means that the 95% CI is not overlapping each other indicating that if a hypothesis test had been conducted the difference would have been significant (p < 0.05)

A randomized sample of physicians in six medical specialties (from all over Sweden) including general practitioners (GPs) (n = 243), geriatricians (n = 243), internists (n = 243), oncologists (n = 244), psychiatrists (n = 240), and surgeons (n = 241) was selected in the 2020 study. The response rate for the 2020 study was approximately 60%. The same medical specialties as in the 2007 study were included, and on average approximately 195 physicians from each specialty received the questionnaire and approximately 70% answered the questionnaire in this study. In the 2020 study, all Swedish palliative care physicians (n = 123) were also included. Palliative medicine only became a medical specialty in 2015 and was accordingly not included as a specialty in the 2007 study, but several participants from other specialties were probably working as palliativists.

In order to examine from which of the traditional specialties the new group of palliativists came, we also compared the two prioritized responses classified as either autonomy based or non-maleficence based for each specialty and compared them with the new group of palliativists. The differences were calculated using Fisher’s exact test (Table 2).

**Table 2:** The differences between palliativists and the six other specialties regarding the number of prioritized autonomy arguments versus the non-maleficence arguments

Specialties	Differences between palliativists (2020) and other specialties in the 2007 study	Differences between palliativists and other specialties in the 2020 study
Difference between GPs and palliativists	p = 0.0003	p = 0.01
Differences between surgeons and palliativists	p = 0.0008	p = 0.002
Differences between geriatricians and palliativists	p = 0.03	p = 0.1
Differences between internists and palliativists	p = 0.07	p = 0.02
Differences between psychiatrists and palliativists	p = 0.08	p = 0.003
Differences between oncologists and palliativists	p = 0.1	p = 0.04
Differences between average of other specialties and palliativists	p = 0.002	p = 0.008

Even though we did not test a hypothesis, we used Fisher’s exact test to calculate a p-value to illustrate the dynamic when palliative medicine became a specialty in 2015 and accordingly decreased the number of participants from other specialties, typically internists and oncologists

In order to better understand the relation between the suggested disguised paternalism index and the attitudes toward PAS in the 2007 and 2020 studies, we also compared the six specialties regarding the specialties’ attitudes (Table 3).

**Table 3:** The results of the six specialties’ attitudes towards physician-assisted suicide regarding the proportions who supported it

Specialties	2007 study	2020 study
GPs (n = 155/141)	37% (29–45)	48% (40–56)
Surgeons (n = 144/138)	39% (31–47)	54% (46–62)
Geriatric (n = 123/129)	33% (25–41)	37% (29–45)
Internists (n = 155/161)	32% (25–39)	44% (36–52)
Psychiatrists (n = 135/127)	41% (33–49)	54% (45–63)
Oncologist (n = 149/145)	26% (19–33)	46% (38–54)*
All (n = 859/841)	35% (32–38)	47% (44–50)*

The results might be compared with the indices presented in Table 4. In comparisons where the 95% confidence intervals did not overlap each other, an * indicates that the differences were significant as if a hypothesis test had been conducted (p < 0.05)

Apart from the indices from the concerned specialties, we also included age groups where respondents < 46 years old were labelled younger physicians, those 46–60 years old as middle-aged physicians, and those > 60 years as older physicians.

The disguised paternalism index was calculated by taking the difference between the number of respondents who argued in favour of respect for patient’s autonomy and that the autonomy principle should precede over the non-maleficence principle (*A*) and the number of respondents who argued that such patients do not know their own best interests and that the non-maleficence principle should take precedence over the autonomy principle (*NM*) and dividing by the number of those who prioritized the *NM* arguments:

$$\frac{\left(A-NM\right)}{NM}$$

The size of the difference determines the size of the index. The smaller the index, the higher the degree of disguised paternalism, and the higher the index the lower the degree of disguised paternalism. If *NM* is larger than *A*, the difference will become negative, which would be a very small index and accordingly a very high degree of disguised paternalism.

Because this index is not representing a fundamental constant, the present index is relative and is dependent on the specific context and hence there are no specific cut-offs for amounts of disguised paternalism. We have used the index as a proxy for the cut-offs of high and low levels of disguised paternalism. The main focus was to compare the indices of the different specialties and different age groups and whether the index changed from 2007 to 2020. In the present text, the dividing line between high and low amounts of disguised paternalism is based on the average index for the respective age group where indices below the average index from the two study samples indicate a high degree of disguised paternalism (Table 4).

**Table 4:** The disguised paternalism indices among the different specialties and age groups for 2007 and 2020

Specialties	Disguised paternalism indices for specialties and age groups 2007	Disguised paternalism indices for specialties and age groups 2020
GPs	(40–17)/17 = 1.35	(54–41)/41 = 0.32
Surgeons	(39–19)/19 = 1.05	(59–36)/36 = 0.64
Geriatricians	(30–25)/25 = 0.2*	(44–45)/45 = − 0.02*
Internists	(31–28)/28 = 0.11*	(63–53)/53 = 0.19*
Psychiatrists	(29–27)/27 = 0.07*	(54–35)/35 = 0.54
Oncologists	(27–26)/26 = 0.04*	(47–41)/41 = 0.15*
Palliativists	−	(15–30)/30 = − 0.5*
*Age groups*		
< 46 years	(59–67)/67 = − 0.12*	(144–99)/99 = 0.45
46–60 years	(96–67)/67 = 0.55	(102–111)/111 = 0.08*
> 61 years	(41–13/13 = 2.2	(94–70)/70 = 0.34
Average (limits)	(196–142)/142 = 0.38	(336–281)/281 = 0.20

When calculating the index, the proportions were from the prioritizing of the arguments. The average indices were used to divide between a high degree of disguised paternalism (< 0.38 and < 0.20) and a low degree of disguised paternalism (> 0.38 and > 0.20). Palliative medicine was established as a specialty in 2015 and therefore not included in the 2007 study

GPs = General practitioners, − = negative index

* indicating high degree of disguised paternalism

The study was approved by the Swedish Ethical Review Authority, Dnr: 2020-01842.

## Results

In order to better understand the suggested and updated indices of the six specialties, we have provided their attitudes towards PAS in terms of those who supported PAS (Table 3). As can be seen, it was only the average proportions between 2007 and 2020 that showed a significant difference [35% (32–38%) versus 47% (44–50%)], and one specialty, oncology also showed a significant difference between 2007 and 2020 [26% (95%CI: 19–33%) versus 46% (95%CI: 38–54%)]. The proportion of palliativists supporting PAS in 2020 was 26% (95%CI: 16–36%).

A comparison of the 2007 results to the 2020 results focusing on how important the participants found the four provided arguments to be, there was only one argument—the open paternalistic argument that such patients do not know their own best interests—with a significant difference; in 2007 it was 74% (95% CI: 71–77%) compared with 59% (95% CI: 55–63%) in 2020 (Table 1). Among palliative care physicians, which were not included in the 2007 study, nobody prioritized the argument that such patients do not know their own best interests, whereas a majority of the palliativists (51%) prioritized the argument that the *non-maleficence principle should overrule the autonomy principle*, 20% prioritized the autonomy argument, and 5% prioritized the argument that *the autonomy principle should overrule the non-maleficence principle*.

The calculated indices for each specialty (and a separate index for palliative care physicians) are presented in Table 4. For the 2007 study, the average index was 0.38, meaning that < 0.38 represented a high degree of disguised paternalism and > 0.38 represented a low degree of disguised paternalism. The corresponding average index for 2020 was 0.20 meaning that indices < 0.20 indicated more disguised paternalism and indices > 0.20 indicated lower levels of disguised paternalism.

Among the palliative care physicians, 20% prioritized the autonomy argument and 5% prioritized the argument that the autonomy principle should overrule the non-maleficence principle.

## Discussion

In the results of the 2007 survey, GPs and surgeons, together with the middle-aged (46–60 years) and the oldest physicians (> 60 years), had low (or in the case of the oldest physicians, very low) degrees of disguised paternalism (that is, their indices were well over the 0.38 average). Geriatricians, internists, psychiatrists, and oncologists had high degrees of paternalism (as indicated by indices less than 0.38), and the youngest age group (< 46 years) had very high degrees of disguised paternalism (as indicated by the negative index value).

The analysis of the results from the 2020 survey revealed a somewhat different pattern of disguised paternalism among the specialties. GPs, surgeons, and psychiatrists had low degrees of disguised paternalism, as did the youngest group of physicians and the oldest group of physicians (all above the 0.20 average). Internists, oncologists, and the middle-aged group were slightly below the average, indicating a high level of disguised paternalism, while only geriatricians and palliativists had negative indices in 2020, indicating that the physicians in these two specialties had quite high degrees of disguised paternalism.

Comparing the 2007 indices to the 2020 indices, we can see that the groups that changed the most in terms of relative amounts of disguised paternalism were geriatricians, psychiatrists, and the youngest physicians. Geriatricians were less paternalistic in the 2007 study than the 2020 study; in contrast, psychiatrists and the group of youngest physicians were more paternalistic in 2007 than 2020.

Comparing the age groups, we found that in 2007 the youngest group of physicians had a high level of disguised paternalism, while the middle-aged and oldest group had low levels. In 2020, the middle-aged group had a high level of disguised paternalism, while the oldest and youngest had low levels. Of course, many individual physicians who were in the youngest group in 2007 had aged into the middle-aged group in 2020. If this trend continues, we might expect that a survey performed in the future—let us say in 2033—would reveal that middle-aged physicians (i.e., today’s young physicians) have a lower degree of disguised paternalism than today’s middle-aged physicians.

When comparing the six specialties and their general attitudes towards PAS (Table 3) with the indices (Table 4), it seems that they are not reflected in each other. This might indicate that the indices represent special results.

### Geriatricians and palliativists

The most relevant result for discussing PAS and other end-of-life issues in health care is the relatively high level of disguised paternalism among geriatricians, and in 2020 even among the palliative care physicians. In 2007, the geriatricians had a high level of disguised paternalism, but in 2020 the index became even lower and negative, indicating a considerably higher level of disguised paternalism. This trend in the geriatric specialty might reflect a reaction to the ongoing covid-19 pandemic, where many elderly and frail patients, sometimes also cognitively impaired, died under conditions that, under normal circumstances, would have meant they would have been offered palliative care [14]. However, even the dysphemism ‘euthanasia’ was used in the Swedish debate when discussing elderly patients with covid-19 [15]. The majority of these patients would not have been referred to emergency hospitals under normal conditions, but during the pandemic not treating patients suffering from covid-19 pneumonia was considered to be a type of assisted dying [15]. The use of terms like ‘palliative care’ and ‘euthanasia’ might have influenced geriatricians and their attitudes towards the PAS issue and stressed that the ‘non-maleficence principle should precede the autonomy principle’ when weighing it against the autonomy principle. Some of the geriatric patients may have been physically frail with several comorbidities, but still mentally competent, and we do not know what they would have preferred. Sometimes elderly frail patients actually abstain from treatment even though the risk of treatment is negligible but the consequences are fatal [6]. Some of these patients might also have a dementia diagnosis, and it is possible that geriatricians were trying to protect these non-competent patients by applying soft and justifiable paternalism.

The other specialty with very high disguised paternalism index was palliativists. Comparing the response pattern of palliativists and the six specific specialties and calculating p-values allows us to say something about from which specialties the palliativists come. Internal medicine, psychiatry, and oncology are the three specialties where the p-values decreased—indicating a more significant difference (Table 2). Because palliativists are the specialty with the highest level of disguised paternalism and they disappear from a certain specialty, the disappearance might have some impact on the actual specialty making it less paternalistic. The number of palliativists was relatively low and the impact might have been limited, and there might also have been other explanations for the increased differences—e.g. that internists, oncologists, and psychiatrist tend to be less paternalistic.

Moreover, palliative care physicians take care of many suffering patients at the end of life, and it seems reasonable to ask whether this disguised paternalism influences the decisions made by these physicians. There are indications that palliativists’ personal values influence whether or not they are prepared to apply continuous deep sedation [3, 4], which is particularly relevant because ‘good palliative care’ together with palliative sedation is often claimed to be an adequate or even better alternative to PAS [13]. An international trend is that palliativists are currently more inclined to provide continuous deep sedation on a patient’s request [16, 17]. However, this trend is not yet observable in Sweden, which is problematic because continuous deep sedation on request could in many cases replace PAS—although not in all cases [18]. Some palliativists seem to have not only disguised paternalism, but also the form of paternalism in which they claim that they are *protecting* or *preserving* a patient’s autonomy rather than *respecting* it [3, 19]. Because such palliativists are referring to autonomy, their attitude becomes paternalistic in the name of autonomy, which is a sophisticated form of disguised paternalism [20]. In practice, a patient might request continuous deep sedation, but in order to protect or preserve the patient’s autonomy superficial and intermittent palliative sedation is provided instead, even against the patient’s preferences and wishes [5]. Such a patient might be brought to consciousness up to four times per day in order to be asked whether or not they would like to continue being sedated [3, 4]. Representatives from the general public have described such procedure as macabre or cruel and inhumane [21].

As can be seen from these clinical examples, disguised paternalism might have serious consequences for patients, especially within palliative care. If such patients are truly competent, their wishes and preferences at the end of life should be respected.

The 2020 study indicated that only one in four palliativists supported PAS, which still leaves a clear majority against PAS, and it has been shown that palliativists’ personal values influence their practice and their regard for patients’ wishes [3, 4, 9]. Disguised paternalism is not solely an academic issue—it has consequences when equal cases are not treated equally—and, accordingly, disguised paternalism should be revealed when possible and counteracted.

As stated in the introduction, the term disguised paternalism might also include the physician’s disguised values and preferences as well as disguised self-interest. However, the present study with the suggested index does not make it possible to separate these entities, and this is a limitation that future studies might examine more closely.

## Strengths and limitation

Several more fixed options for and against PAS were presented in the 2007 survey than the 2020 survey, which meant that we had to eliminate 275 responses when recalculating the 2007 results to compare with the 2020 results. The 2007 study used two combined arguments, ‘non-maleficence should overrule the autonomy principle argument’ and ‘the patient does not know her/his own best interests’, and we subtracted these collapsed numbers from all other arguments and accordingly the difference became somewhat higher (compared to the 2020 study) and accordingly also the average index. Using the average index as the boundary for each separate study makes it somewhat easier to compare the two studies.

It should be stressed that the indices are associated with the PAS issue, and therefore the results should not be generalized to other areas and issues. The average index would fluctuate based on the participants and circumstances, but would probably systematically change depending on when the survey is done and how controversial the main issue is. Moreover, it is important to stress that the average index should be considered as a proxy for discrimination between high and low levels of disguised paternalism. Previously conducted studies indicated that the influence of physicians’ personal values and preferences are more expressed on issues that are more controversial, and PAS is a controversial issue, at least in the Swedish medical context [22]. Moreover, in Sweden there is no such thing as conscientious objection and accordingly physicians might have reasons for hiding their personal values and preferences, their self-interests, and their paternalistic behaviour towards patients—aspects that are against the Swedish health care law.

## Conclusions

This study suggests that the proposed index might be useful as an indicator of disguised paternalism regarding competent patients and that disguised paternalism might be present when reasoning about a controversial issue such as PAS. If we assume that the suggested average indices might be used as proxies for discriminating between high levels and low levels of disguised paternalism, this tells us is that disguised paternalism is quite common among certain age groups of physicians as well as certain specialties. It should, however be stressed that the actual indices were developed within the controversial issue of PAS and should not be generalized to less controversial issues.

Overall, there was less open paternalism in the 2020 study than the 2007 survey, but going from open paternalism towards less open paternalism might indicate that disguised paternalism may be seen as more acceptable due to the fact that it may look like something else. Palliativists, who are the physicians actually treating suffering patients at the end of life, showed the highest degree of disguised paternalism, which could have implications for when and if continuous deep palliative sedation is offered. Disguised paternalism or disguised personal values or self-interest might jeopardize the patient’s right to shared decision-making. Accordingly, it is important to further improve the suggested index for disguised paternalism and to develop other methods of approaching this delicate issue within less controversial issues.

## Data Availability

The datasets generated and analysed during the current study are available from the corresponding author on reasonable request.

## Code availability

The WHO-based software program Epi-Info was applied when performing the registration and analysis of data. The data from this study might be made available by contacting the corresponding author.
